# Supplementary material for: Sex Differences in the Anti-Hypertensive Effect of Calcium-Channel Blockers: A Systematic Review and Meta-Analysis
Source: Biomedicines. 2023 Jun 2;11(6):1622. doi: 10.3390/biomedicines11061622 (PMC10296139; doi:10.3390/biomedicines11061622)
Supplement: Supplementary file 1 [file biomedicines-11-01622-s001.zip › biomedicines-2402809-supplementary.docx]

**SUPPLEMENTS**

| **Table S1** Literature search: strategy for PubMed (NCBI) and Embase (Ovid) databases | |
| --- | --- |
| **Search PubMed** | **Search Embase** |
| **Component 1: Antihypertensive medication:** "diuretics"[Mesh] OR "adrenergic beta-antagonists"[Mesh] OR “beta blockers” [Title/Abstract] OR "Antihypertensive agents"[Mesh] OR “blood pressure lowering therapy” [Title/Abstract] OR “antihypertensive medication” [Title/Abstract] OR “antihypertensive therapy” [Title/Abstract] OR "angiotensin-converting enzyme inhibitors"[Mesh] OR “ACE inhibitors” [Title/Abstract] OR "Angiotensin receptor antagonists"[Mesh] OR “angiotensin receptor blockers” [Title/Abstract] OR "sympatholytics"[Mesh]OR "Calcium Channel Blockers"[Mesh] | **Component 1: Antihypertensive medication**  exp diuretic agent/ or exp beta adrenergic receptor blocking agent/ or exp adrenergic receptor blocking agent/ or exp antihypertensive agent/ or exp dipeptidyl carboxypeptidase inhibitor/ or exp angiotensin receptor antagonist/ or exp calcium channel blocking agent.ti,ab. |
| **Component 2: Cardiac geometry:** "ventricular remodeling"[Mesh] OR “ventricular remodeling” [Title/Abstract] OR “cardiac remodeling” [Title/Abstract] OR “cardiac adaptation” [Title/Abstract] OR “LV geometry” [Title/Abstract] OR “left ventricular geometry” [Title/Abstract] OR “cardiac geometry” [Title/Abstract] OR “cardiac dimension” [Title/Abstract] OR "left ventricle remodeling "[ Title/Abstract] OR " Hypertrophy, Left Ventricular "[Mesh] OR “left ventricular hypertrophy” [Title/Abstract] OR "echocardiography"[Mesh] OR Echocardiography [Title/Abstract] OR “left ventricular mass” [Title/Abstract] OR “left ventricular mass index” [Title/Abstract] OR “relative wall thickness” [Title/Abstract] OR “concentric cardiac remodeling” [Title/Abstract] OR “eccentric cardiac remodeling” [Title/Abstract] | **Component 2: Cardiac geometry**  exp heart ventricle remodeling/ or (ventricular remodeling or cardiac remodeling or cardiac adaptation or LV geometry or left ventricular remodeling or cardiac geometry or cardiac dimension).ti,ab. or exp echocardiography/ or echocardiography.ti,ab. |
| **Component 3: Heart failure:** "Heart Failure"[Mesh] OR "Heart Failure, Systolic "[Mesh] | **Component 3: Heart failure**  exp heart failure.ti,ab. |
| **Component 4: Diastolic dysfunction:** “heart failure, diastolic”[Mesh] OR “diastolic dysfunction” [Title/Abstract] | **Component 4: Diastolic dysfunction**  exp diastolic dysfunction/ or diastolic function.ti,ab. |
| **Component 5: Myocardial infarction:** "myocardial infarction" [Mesh] OR “myocardial infarction” [Title/Abstract] OR “acute myocardial infarction” [Title/Abstract] OR “heart attack” [Title/Abstract] | **Component 5: Myocardial infarction**  exp heart infarction.ti,ab. |
| **Component 6: CVA**: Stroke [Mesh] OR “cerebrovascular accident” [Title/Abstract] OR “acute cerebrovascular accident” [Title/Abstract] OR “acute cerebrovascular insult” [Title/Abstract] | **Component 6: CVA**  exp cerebrovascular accident.ti,ab. |

Combination search terms: component 1 AND (component 2 OR component 3 OR component 4 OR component 5 OR component 6).

**Table S2** Chemical-pharmacological distinction dihydropyridines (D) and non-dihydropyridines (ND)

**Table S2.1** Pooled changes in cardiovascular and haemodynamic parameters for females and males using dihydropyridines

| Parameter |  | Females (D) | Males (D) |
| --- | --- | --- | --- |
| SBP (mmHg) | MD  % | -25.4 (-34.3; -16.6)  - 15.8 (-21.2; -10.3) | -19.9 (-23.8; -16.1)  -12.9 (-15.4; -10.5) |
| DBP (mmHg) | MD  % | -13.4 (-18.4; -8.4)  -14.5 (-19.9; -9.1) | -13.4 (-16.1; -10.7)  -14.2 (-17.1; -11.3) |
| MAP (mmHg) | MD  % | -13.9 (-14.5; -13.2)  -12.5 (-13.1; -12.0) | -8.7 (-14.1; -3.3)  -8.9 (-14.5; -3.4) |
| HR (bpm) | MD % | -1.9 (-2.5; -1.2)  -2.5 (-3.4; -1.6) | 2.2 (0.0; 4.4)  3.0 (0.0; 6.0) |
| CO (L/min) | MD % | -0.2 (-1.8; 1.4)  -4.0 (-35.8; 27.7) | 0.9 (0.1; 1.7)  19.3 (1.9; 36.6) |
| LVEF (%) | MD % | - | 4.3 (1.7; 6.9)  8.2 (3.2; 13.2) |
| LVM (g) | MD % | - | -32.0 (-123.7; 59.7)  - |

Values are reported as mean difference (MD) and relative change (%) compared to baseline with 95% CI.
SBP = systolic blood pressure, DBP = diastolic blood pressure, MAP = mean arterial pressure, HR = heart rate,
CO = cardiac output, LVEF = left ventricular ejection fraction, LVM = left ventricular mass.

**Table S2.2** Pooled changes in cardiovascular and haemodynamic parameters for females and males using non-dihydropyridines

| Parameter |  | Females (ND) | Males (ND) |
| --- | --- | --- | --- |
| SBP (mmHg) | MD  % | -35.6 (-64.5; -6.6)  -22.4 (-40.7; -4.2) | -8.3 (-14.8; -1.9)  -5.9 (-10.5; -1.3) |
| DBP (mmHg) | MD  % | -20.4 (-32.3; -8.6)  -20.7 (-32.9; -8.7) | -5.1 (-9.1; -1.1)  -5.3 (-9.5; -1.1) |
| MAP (mmHg) | MD  % | - | - |
| HR (bpm) | MD % | -0.7 (-5.6; 4.2)  -1.2 (-9.3; 6.9) | -3.1 (-5.1; -1.2)  -4.3 (-7.0; -1.7) |
| CO (L/min) | MD % | - | 0.3 (-1.8; 2.4)  - |
| LVEF (%) | MD % | - 7.8 (-12.4; -3.2) -11.4 (-18.0; -4.7) | -0.4 (-7.1; 6.4)  -0.6 (-11.5; 10.3) |
| LVM (g) | MD % | - | -13.6 (-48.1; 20.9)  - |

Values are reported as mean difference (MD) and relative change (%) compared to baseline with 95% CI.
SBP = systolic blood pressure, DBP = diastolic blood pressure, MAP = mean arterial pressure, HR = heart rate,
CO = cardiac output, LVEF = left ventricular ejection fraction, LVM = left ventricular mass.

**Table S2.3** P-values males vs females distinction dihydropyridines (D) and non-dihydropyridines (ND)

|  | D | ND |
| --- | --- | --- |
| SBP | 0.2622 | 0.0716 |
| DBP | 0.9941 | 0.0162 |
| MAP | 0.0612 | - |
| HR | 0.0005 | 0.3723 |
| CO | 0.2289 | - |
| LVEF | - | 0.0746 |
| LVM | - | - |
